# Patients who underwent total hip or knee arthroplasty are more physically active than the general Dutch population

**DOI:** 10.1007/s00296-016-3598-9

**Published:** 2016-11-16

**Authors:** J. M. T. A. Meessen, W. F. Peter, R. Wolterbeek, S. C. Cannegieter, C. Tilbury, M. R. Bénard, H. M. J. van der Linden, R. Onstenk, R. Tordoir, S. B. Vehmeijer, S. H. M. Verdegaal, H. M. Vermeulen, R. G. H. H. Nelissen, T. P. M. Vliet Vlieland

**Affiliations:** 1 Department of Orthopaedics, Leiden University Medical Center, J11R, P.O. Box 9600, 2300 RC Leiden, The Netherlands; 2 Department of Medical Statistics, LUMC, Leiden, The Netherlands; 3 Department of Clinical Epidemiology, LUMC, Leiden, The Netherlands; 4 Department of Orthopaedics, Reinier de Graaf Groep, Delft, The Netherlands; 5 Department of Orthopaedics, Groene Hart Ziekenhuis, Gouda, The Netherlands; 6 Department of Orthopaedics, Rijnland Ziekenhuis, Leiderdorp, The Netherlands; 7 Department of Physiology, LUMC, Leiden, The Netherlands; 8 Rijnlands Rehabilitation Centre, Leiden, The Netherlands; 9 Sophia Rehabilitation, The Hague, The Netherlands

**Keywords:** Osteoarthritis, Physical activity, Arthroplasty, THA, TKA

## Abstract

Total hip arthroplasty (THA) and total knee arthroplasty (TKA) bring relief of pain and functional disability to patients with end-stage osteoarthritis, and however, the literature on their impact on patients’ level of physical activity (PA) is scarce. Cross-sectional study in patients who underwent THA/TKA surgery in the preceding 6–22 months and a random sample of persons aged >40 years from the Dutch general population, participating in a national survey. PA in minutes per week (min/week) and adherence to the Dutch recommendation for PA (NNGB yes/no) were measured by the short questionnaire to assess health-enhancing PA. Multivariable linear (total min/week) and logistic regression analyses (meeting recommendations PA), adjusting for confounders, were performed for THA and TKA separately. In total, 258 THA [62.3% female, aged 69.4 (9.1)] and 221 TKA [65.7% female, aged 69.5 (8.9)] patients and 4373 persons from the Dutch general population [51.4% female, aged 58.9 (11.6)] were included. The presence of THA was associated after adjusting for age, sex, BMI education and musculoskeletal comorbidities, with more total min/week spent on PA (THA 13.8% increase, 95% CI 1.6–27.6%), whilst both TJA groups were associated with adhering to NNGB (THA: OR 1.79, 95% CI 1.26–2.56; TKA: OR 1.73, 95% CI 1.20–2.51). As this study used questionnaires to compare the PA of THA/TKA patients to the general population, some recall and selection bias might have been induced. After surgery, overall, TJA patients are more likely to adhere NNGB than a representative sample of persons >40 years from the Dutch general population.

## Introduction

Worldwide, the numbers of patients undergoing total hip or total knee arthroplasty (THA or TKA) for hip or knee osteoarthritis (OA) are rapidly increasing. Overall, the outcomes are favourable, with a large majority of patients having less pain and improved physical functioning after surgery [1–4].

Although the benefits of THA and TKA are well documented for pain and function, relatively little is known on their impact on one specific aspect of physical functioning, i.e. physical activity (PA). Just like for any other individual, achieving and maintaining a sufficient level of PA is important for patients with hip and knee OA with respect to their potential general health benefits. Moreover, in patients who undergo THA or TKA, PA may have an additional beneficial effect on the quality of the bone, which in turn may prevent complications such as early loosening [5–8]. In addition, PA may have a positive effect on muscle strength and range of motion of the affected leg [9].

With respect to the literature on PA after THA or TKA, de Groot et al. demonstrated in 84 THA and TKA patients that 6 months post-operatively PA levels as measured with an activity monitor did not significantly differ from the preoperative activity levels [10]. Harding et al. [11] found similar results when measuring PA by means of an accelerometer in 63 American THA and TKA patients before surgery and 6 months post-operatively. Kahn and Schwartzkopf [12] found no difference in PA as measured with an accelerometer between those on the waiting list for TKA and those who had TKA 2 years earlier.

By using the patient-reported University of California at Los Angeles (UCLA) activity questionnaire, Baumann et al. [13] found that both THA and TKA patients were regularly active on moderate to high levels after on average 6–12 months after surgery. This finding is supported by Dahm et al. [14], who reported that 5.7 years after surgery TKA patients had an average physical activity score of 7.1 out of 10, with 10 being highly active. In contrast to these findings, Kahn and Schwartzkopf [12] observed, using an accelerometer, that adherence to health-enhancing PA guidelines was only 5% in persons with TKA. In all of these studies, a comparison with the general population was lacking.

Two Dutch studies compared patient-reported physical activity in THA [15] and TKA [16] patients at 1–5 years post-operatively to that of age and gender-matched controls. It was found that in THA the proportion of persons reaching the Dutch Public Health Physical Activity guideline (the “Nederlandse Norm Gezond Bewegen”, NNGB) was similar to that of matched controls [51.2% (THA) vs 48.8% (controls)] [15], whereas in TKA patients the proportion of patients adhering to the guideline (54.5%) was significantly lower than that of the matched control population (63.7%) [16]. However, these studies did not take BMI into account, whilst BMI is one of the determinants of physical activity [17].

Given the lack of knowledge on post-operative PA levels after total joint arthroplasty (TJA) compared to the general population, the aim of the present study was to compare the minutes of PA and proportion meeting the public health guidelines of THA and TKA patients to those of the general Dutch population. Moreover, factors other than TJA, possibly contributing to levels of physical activity, were also evaluated.

## Patients and methods

### Design and recruitment of subjects

This cross-sectional, multicentre study concerned a comparison of PA levels of THA and TKA patients approximately one year after surgery with those of the general Dutch population. The data from the population of patients with THA and TKA were obtained from a study primarily aiming to make an inventory of the use of physical therapy and the presence of comorbidity [18], whereas the data from the general population were obtained from the Dutch National Bureau of Statistics (in Dutch: Centraal Bureau voor de Statistiek, CBS).

Since the survey had to be filled in only once by patients, it was judged to fall outside the remit of the law for Medical Research Involving Human Subjects Act; MO [in Dutch; Wet medisch wetenschappelijk onderzoek met mensen (WMO)]. An exemption for medical ethical review was therefore given by the Medical Ethical Committee of the Leiden University Medical Center. The health monitoring conducted by the CBS commissioned by the Dutch Government also falls outside the remit of the WMO. The study was conducted in accordance with the Handbook for Good Clinical Research Practice of the World Health Organization [19] and the Declaration of Helsinki principles [20].

### Patients with THA or TKA

The patient data were obtained from a cross-sectional study performed in 2012, including patients who underwent THA or TKA for hip or knee OA in 2011 in four different hospitals in the Leiden region (Leiden University Medical Center in Leiden, Rijnland Hospital in Leiderdorp, Groene Hart Hospital in Gouda and Reinier de Graaf Hospital in Delft, the Netherlands). Patients receiving THA or TKA for reasons other than end-stage OA (such as fracture or rheumatoid arthritis) were excluded from the study, as well as patients undergoing revision surgery.

Between July 2012 and October 2012, all patients operated in 2011 were approached by mail by their orthopaedic surgeon, resulting in a range of post-surgery time of 7–22 months. The orthopaedic surgeon sent all eligible persons an invitation letter, information leaflet, informed consent form, survey and pre-stamped return envelope. Patients who returned the envelope with a completed survey and signed informed consent were included in the study.

### Data general population

Data from the Dutch general population were provided by the CBS and were derived from a nationwide survey on general health (Gezondheids-enquête) [21]. This questionnaire is annually administered to a representative sample of ±8.000 Dutch inhabitants and is the prime health monitor tool of the Dutch government [22].

The selection of participants is drawn from municipality registers. Persons living in institutionalized homes (e.g. nursing homes) are excluded. For the present study on physical activity, only data were selected from 2011, i.e. the same year as the data from patients with THA or TKA, and from respondents who were over 40 years of age, as none of the persons with arthroplasty was aged below 40.

### Assessments

Included in both surveys were the following variables or questionnaires.

#### Socio-demographic and basic health characteristics

Demographic variables included: age, gender and marital status (split into either married or not married). The height and weight of the patient were asked in order to calculate the body mass index (BMI). Smoking status (non-smoker, ex-smoker and smoker) and educational level (low (elementary school, lower secondary education), medium (secondary school or college) or high (higher secondary education or university)) were recorded.

#### Physical activity

PA was assessed using the validated Dutch version of the short questionnaire to assess health (SQUASH) [23, 24]. The SQUASH records the total amount of minutes per week (min/week) spent on PA in an average week in the past 12 months regarding eight different domains of active life: commuting, work activities, walking, cycling, gardening, odd jobs, household and sports. With the aid of the compendium of Ainsworth [25], PA can subsequently be categorized into light, moderate or vigorous intensity. Using this information, it is possible to define whether an individual adhered to the Dutch Public Health recommendation (NNGB) for PA (30 min of moderate intensity PA on at least 5 days per week) [26].

#### Quality of life (QoL)

QoL of the persons with a THA/TKA was assessed with the Short Form 36 (SF36) questionnaire, whilst the QoL of the general Dutch population was assessed with the Short Form 12 (SF12) [27, 28]. The SF36 outcomes of the THA/TKA patients were transformed to SF12 outcomes. From the SF12, two summary scales were derived: the physical component scale (PCS) and the mental component scale (MCS). The higher the score on these scales, the better the physical or mental functioning.

#### Comorbidity

The presence of comorbidity was assessed by means of a self-reported questionnaire of the CBS which comprised 19 different comorbidities [29]. For every comorbidity, the participants of the survey were asked to respond with either yes or no to the question “Have you received any treatment for [condition] in the past year”. The included diseases were then clustered into three groups:

##### Musculoskeletal comorbidities

Severe back pain (including slipped disc), severe neck or shoulder pain, severe elbow wrist or hand pain, inflammatory arthritis or other joint conditions.

##### Non-musculoskeletal comorbidities

Asthma or COPD (chronic obstructive pulmonary disease), (severe) cardiac disorder or coronary disease, arteriosclerosis (abdomen or legs), hypertension, (consequences of) stroke, severe bowel disorder, diabetes mellitus, migraine, psoriasis, chronic eczema, cancer and urine incontinence.

##### Sensory comorbidities

Hearing impairments (group and face-to-face conversation), vision impairments (short and long distance) and dizziness in combination with falling.

### Statistical analyses

The demographic and health characteristics of patients undergoing THA or TKA were each compared with those of the general Dutch population by means of two sample *t* tests or Chi-square tests, where appropriate.

Mann–Whitney tests were conducted to compare the min/week spent on PA for each of four different age groups (aged under 65, 65–69, 70–74 and 75+).

The min/week of PA was log-transformed to reach a normal distribution. Multivariable linear regression models were used to assess whether having had a joint replacement was associated with min/week spent on PA. Each analysis was done separately for THA versus the general Dutch population and TKA versus the general Dutch population. The antilog of the effect sizes (beta’s) is reported for both the analyses with the 95% confidence interval (CI).

Multivariable logistic regression was used to assess the association between the presence of a joint replacement and adherence to the Dutch public health physical activity guideline. These results are presented as odds ratio (OR) with the 95% CI. The analyses were done separately for THA versus the general Dutch population and TKA and the general Dutch population.

All models (multivariable linear and multivariable logistic regression analyses) were constructed using a stepwise method. Potential confounders for the level of physical activity, i.e. sex, BMI, age and education level, were included in the models.

The determinants of minutes per week spent on activities categorized according to the three different levels of intensity of physical activity were determined for the arthroplasty groups and the Dutch population separately, by means of linear regression models including the variable of interest and correcting for age and sex. These analyses were performed including the variables age, sex, BMI, education, non-musculoskeletal comorbidities, musculoskeletal comorbidities, sensory comorbidities, MCS, PCS and time since surgery.

The level of statistical significance was set at *P* < 0.05, and analyses were performed using the SPSS statistical package (version 20.0, SPSS, Chicago, IL).

## Results

### Study population

Of the 545 THA and 465 TKA patients of the 4 hospitals who were invited to participate, 258 THA patients (response rate 47.3%) and 221 TKA patients (response rate 47.5%) completed the questionnaires.

The selection of data from the general Dutch population from the year 2011 yielded 4373 surveys completed by people aged 40 years or older. Of those, 568 persons (13%) replied positively to the question “Have you received any treatment for osteo- or rheumatic arthritis in the past year?”.

The arthroplasty groups comprised statistically significantly more females, and the patients had a higher mean age and higher BMI than the general Dutch population. The PCS and MCS were statistically significantly lower in both the THA and the TKA groups than in the general population. There was no difference in the presence of sensory comorbidities between the arthroplasty groups and the general population. However, both musculoskeletal and non-musculoskeletal comorbidity were more present in the arthroplasty patients as compared to the general population (see also Table 1).

**Table 1:** Characteristics of patients with total joint arthroplasty and a sample from the general Dutch population

		General Dutch population			Total hip arthroplasty				Total knee arthroplasty			
								*P* value^*α*^				*P* value^*α*^
Total	#	4373			258			–	221			–
Female	# (%)	2248	(51.4%)		159	(62.3%)		0.01	144	(65.7%)		<0.01
Age (years)	Mean (SD)	59	(11.6)		69	(9.1)		<0.01	70	(8.9)		<0.01
BMI	Mean (SD)	26	(5.0)		27	(4.1)		<0.01	29	(5.0)		<0.01
Education level								<0.01				<0.01
Low education	# (%)	653	(15.6%)		75	(37.3%)			72	(41.1%)		
Medium education	# (%)	2391	(57.0%)		86	(42.8%)			81	(46.3%)		
High education	# (%)	1150	(27.4%)		40	(19.9%)			23	(12.6%)		
SF12												
PCS	mean (SD)	53.9	(9.4)		47.6	(11.2)		<0.01	46.6	(10.8)		<0.01
MCS	mean (SD)	44.1	(5.0)		39.8	(5.2)		<0.01	40.6	(5.1)		<0.01
Comorbidities												
≥1 Non-M.S^a^	# (%)	2465	(57.4%)	(*N* = 4292)	142	(72.8%)	(*N* = 195)	<0.01	138	(84.1%)	(*N* = 164)	<0.01
≥1 M.S.^b^	# (%)	1224	(27.9%)		93	(39.7%)	(*N* = 238)	<0.01	84	(40.7%)	(*N* = 206)	<0.01
≥1 Sensory	# (%)	400	(9.3%)	(*N* = 4319)	21	(8.4%)	(*N* = 249)	0.736	21	(9.9%)	(*N* = 212)	0.717

Education levels: low (elementary school, lower secondary education); medium (secondary school or college); and high (higher secondary education or university)

*BMI* body mass index, *SF12* Short Form 12, *PCS* physical component subscale of the SF12, *MCS* mental component subscale of the SF12

^*α*^ *P* value is given for a two sample *t* test or Chi-square between the hip or knee replacement group and the general Dutch population

^a^Non-musculoskeletal comorbidities

^b^Musculoskeletal comorbidities

**Fig. 1:**
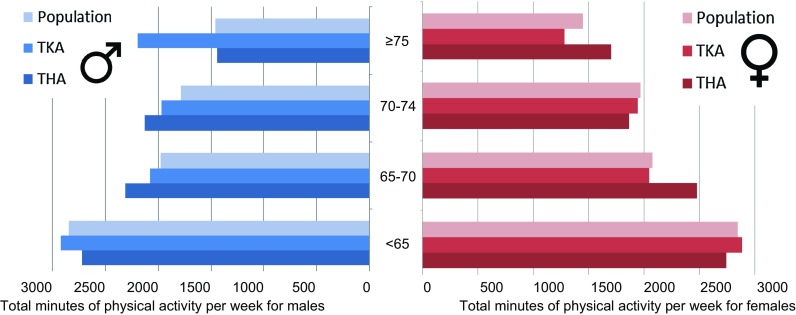
Stratified representation of total minutes per week physical per week per age group and gender

Table 2 shows the crude number of minutes spent per week on PA, stratified for age and gender. It can be seen that the male arthroplasty patients spend more minutes per week physically active in the higher age groups as compared to the general Dutch population. The proportion of persons adhering to the NNGB guideline is in the arthroplasty groups higher than in the general Dutch population (THA 76%, TKA 73% and general Dutch population 68%) (Fig. 1).

**Table 2:** Total minutes per week spent on physical activity per gender, age group for total hip arthroplasty, total knee arthroplasty or general Dutch population

	General Dutch population			Total hip arthroplasty				Total knee arthroplasty			
	Mean	SD	*N*	Mean	SD	*N*	*P* value^α^	Mean	SD	*N*	*P* value^β^
Men											
Aged <65	2846	1543	2521	2722	1252	32	0.692	2924	1468	21	0.959
Aged 65–69	1976	1474	414	2315	1336	23	0.052	2079	1316	20	0.34
Aged 70–74	1784	1330	313	2130	843	20	0.003	1968	1667	12	0.912
Aged ≥75	1461	1209	372	1443	1065	21	0.840	2193	1477	22	0.018
Women											
Aged <65	2845	1530	2761	2745	1148	38	0.857	2883	1695	46	0.956
Aged 65–69	2075	1412	401	2480	1908	34	0.364	2048	1539	25	0.764
Aged 70–74	1967	1273	286	1867	1071	33	0.888	1944	2122	28	0.239
Aged ≥75	1449	1173	525	1703	1453	53	0.350	1284	912	45	0.577

	Count	%	*N*	Count	%	*N*	*P* value^*δ*^	Count	%	*N*	*P* value^*ε*^
Adherence to NNGB	2954	67.6	4373	195	75.6	258	0.007	161	72.9	221	0.105

*NNGB* Nederlandse Norm Gezond Bewegen, Dutch public guideline for physical activity

^*α*^ *P* value for Mann–Whitely test between the hip arthroplasty group and the general Dutch population

^*β*^ *P* value for Mann–Whitely test between the knee arthroplasty group and the general Dutch population

^*δ*^ *P* value for Chi-square test between hip arthroplasty group and the general Dutch population

^*ε*^ *P* value for Chi-square test between knee arthroplasty group and the general Dutch population

### Association between TJA and minutes per week spent on total physical activity

Univariately, both THA and TKA were significantly associated with more minutes per week physical activity when compared to the general population. As there were major differences between the groups, it was needed to correct for potential confounders. When correcting for age, gender, BMI, education and musculoskeletal comorbidities a statistically significant association was for THA, not TKA. With the adjustments, persons with a THA spend 13.8% more minutes per week on physical activity compared to the general population (see Table 3).

**Table 3:** Regression analyses of min/week and adherence to NNGB

	Total hip arthroplasty				Total knee arthroplasty			
	*B*	95% CI		*P* value	*B*	95% CI		*P* value
Total min/week PA								
Univariate^a^	1.229	1.099	1.352	<0.001	1.324	1.186	1.483	<0.001
Multivariate 1^b^	1.14	1.023	1.274	0.018	1.122	0.998	1.262	0.055
Multivariate 2^c^	1.138	1.016	1.276	0.024	1.112	0.986	1.256	0.084
Adherence to NNGB								
Univariate^d^	1.487	1.111	1.989	0.008	1.289	0.952	1.745	0.101
Multivariate 1^e^	1.75	1.243	2.465	0.001	1.75	1.219	2.512	0.002
Multivariate 2^f^	1.789	1.253	2.556	0.001	1.731	1.195	2.507	0.004

^a^Univariate analysis of total min/week PA ~ arthroplasty

^b^Multivariate analysis of total min/week PA ~ arthroplasty + age + sex + BMI + education

^c^Multivariate analysis of total min/week PA ~ arthroplasty + age + sex + BMI + education + musculoskeletal comorbidities

^d^Univariate analysis of adherence to NNGB ~ arthroplasty

^e^Multivariate analysis of adherence to NNGB ~ arthroplasty + age + sex + BMI + education

^f^Multivariate analysis of adherence to NNGB ~ arthroplasty + age + sex + BMI + education + musculoskeletal comorbidities

### Association between TJA and meeting public health recommendation

The multivariable logistic regression models showed that, adjusted for age, gender, BMI, education and muscular comorbidities, both THA and TKA patients had a significantly higher likelihood of meeting public health recommendations for healthy PA as compared to the general population [THA: OR 1.79 (95% CI 1.25–2.55); TKA: OR 1.73 (95% CI 1.20–2.51)]. See also Table 3.

### Determinants of physical activity

Regarding the determinants of physical activity in the general population, age, sex, PCS and education were found to be statistically significantly associated with total minutes per week spent on PA and per category of intensity (see Table 4). In the general population, BMI was associated with the number of min/week of moderate and vigorous intensity PA, but not with light intensity PA. Within both the arthroplasty groups, it was found that age was a significant determinant of total min/week of PA and the min/week of light intensity PA. Within the THA group, age was also a determinant for the min/week of moderately intensive PA. Sex was associated with the min/week of moderate and vigorous intensity PA in both arthroplasty groups and also with the min/week of light intensity PA in the THA group.

**Table 4:** Variables statistically significantly associated with min/week physical activity after correcting for age and sex

General Dutch population			THA			TKA		
	Beta	*P*		Beta	*P*		Beta	*P*
Total physical activity								
Age	0.973	<0.001	Age	0.966	<0.001	Age	0.966	<0.001
Sex^a^	0.953	0.037						
BMI	0.995	0.022	BMI	0.960	0.005			
PCS	1.016	<0.001						
Low education^b^	0.774	<0.001						
Non-M.S. comorbidities^c^	1.076	0.002						
M.S. comorbidities^d^	1.052	0.046						
Sensory comorbidities^e^	1.202	<0.001	Sensory comorbidities^e^	1.517	0.028			
Light physical activity								
Age	0.966	<0.001	Age	0.604	<0.001	Age	0.973	<0.001
Sex^a^	0.748	<0.001	Sex^a^	0.604	<0.001			
						BMI	0.759	0.044
PCS	1.007	<0.001						
Low education^b^	0.809	<0.001						
Medium education	0.834	<0.001						
Moderate physical activity								
Age	0.991	<0.001	Age	0.984	0.031			
Sex^a^	1.084	0.02	Sex^a^	1.596	0.001	Sex^a^	1.538	0.009
BMI	0.912	0.022				BMI	0.964	0.027
PCS	1.016	<0.001						
MCS	0.989	0.001						
Low education^b^	1.180	0.006						
Medium education	1.384	<0.001						
Vigorous physical activity								
Age	1.026	<0.001						
Sex^a^	1.189	<0.001	Sex^a^	1.489	0.005	Sex^a^	1.432	0.022
BMI	0.986	0.003						
PCS	1.014	<0.001				PCS	1.021	0.027
						MCS	0.957	0.008
Medium education^b^	1.151	0.002						

^a^Females were reference

^b^High education as reference

^c^Affected with non-musculoskeletal comorbidities persons as reference

^d^Affected with musculoskeletal comorbidities persons as reference

^e^Affected with sensory comorbidities persons as reference

In the general population, comorbidities were only found to be a determinant of total minutes per week of PA, but not of the min/week in the three categories of PA intensity. In THA patients only the presence of sensory comorbidities was associated with the total min/week of PA, whereas in TKA the presence of comorbidities was not associated with PA.

Although the association of a number of potential determinants with PA in the TKA and TJA groups did not reach statistical significance, overall the directions of the associations were similar to those within the general population (results not shown).

## Discussion

This study demonstrated that the presence of a THA was associated with more min/week spent on PA as well as better adherence to public health recommendations for PA (NNGB) when compared to the general population. TKA was found to only be associated with adhering to the NNGB when compared to the general Dutch population.

Overall, it seems the Dutch population spends more minutes per week on physical activity, but since the patient group differs from the general population the comparison between these groups should be adjusted. When adjusting for age, sex, BMI and education it is found that persons with THA do spend more minutes per week on physical activity and that persons with a THA and TKA are more likely to adhere to the Dutch guideline on physical activity, NNGB. That TKA is associated with the NNGB but not to the minutes per week activity can be explained by the level of intensity of the physical activity performed.

In the general population, more associations between potential determinants of physical activity and the actual numbers of PA reached statistical significance than in the arthroplasty groups. The lack of significance is probably due to the relatively small sample sizes in the arthroplasty groups, limiting the statistical power.

Our groups spent more min/week on PA than reported by two other Dutch studies (for THA in this study 2183 min/week PA, THA in Wagenmakers et al. 1601 min/week, for TKA in this study 2153 min/week PA, TKA in Kersten et al. 1347 min/week PA) [15, 16]. In parallel, regarding the proportion of patients adhering to the Dutch recommendation for physical activity, the outcomes were more favourable in the present study (THA in this study 75.6% and THA in Wagenmakers et al. 51.2%; TKA in this study 72.8% and TKA in Kersten et al. 55%).

Both these latter two studies were done at 1–5 years post-surgery, and our study included patients within the first 22 months after surgery. As reported earlier by our group (Peter et al.), 43.5% of the THA patients and 50.5% of the TKA patients had post-operative physiotherapy for more than 3 months [30]. This implies that a vast amount of our patients might still have intense training with aid of physiotherapists, motivating patients to adhere to the PA. As for the other two studies (Kersten, Wagenmakers), no data on prolonged post-operative physiotherapy are present, and thus, these patients might resume easier into their old, less active activity level.

A recent systematic review on physical activity after THA or TKA measured with accelerometers showed that the post-operative PA levels were lower in the arthroplasty groups as compared to healthy control participants [31]. The differences in outcome could be because our sample of the general population might not be totally healthy and be less active than selected healthy persons. Also, as this study used a questionnaire whilst the systematic review concerned objective measures, participants might have caused some recall bias.

The general population in our study had an adherence rate to the Dutch PA of 67.5% which is comparable to reports from CBS published (Dutch adult population, 66% adhered to the Dutch public health physical activity guideline in 2012) [32]. The minutes per week spent on PA in our study was also consistent with the numbers reported by CBS (2589 min per week in 2012 and 2525 min per week in our study for overall physical activity for the Dutch population) [33].

Factors we identified as influencing the level of PA of persons with hip or knee arthroplasty (BMI, increased age, physical component score) are in line with the findings in a systematic review by Stubbs et al. [34] regarding PA in patients with hip or knee OA. The inverse association of BMI on the level of PA shows that it is an important factor, as well as age and gender, to include in any case–control study [17].

Low-impact activities like walking or cycling seem to protect against function loss and experienced pain from OA [35, 36], in contrast heavy load activities might be a risk factor for the development of osteoarthritis, but also early implant failure although debate exists on the latter [37, 38]. Since contradictory evidence exists on this topic, research into this field is necessary.

Current post-operative rehabilitation after a hip or knee arthroplasty is focussed at independent ambulation and regaining a normal walking pattern, which was deteriorated in the years before surgery due to the slowly progressing osteoarthritis. Secondary to this it aims at getting physically active patient. As mentioned before, about half of our patients reported to receive physiotherapy for more than 3 months after surgery [30]. This might imply that these patients are more motivated to be active than the general population.

Another reason for the higher levels of PA in the arthroplasty groups might be the fact that PA is a risk factor for TJA [39]. As shown by de Groot et al. [10], the post-operative levels of PA did not significantly differ from preoperative levels, suggesting that PA levels of TJA were probably higher than those of the general population before surgery as well.

Finally, the patients filling in the questionnaire knew that the subject of the study was PA, whilst the Dutch general population had to fill in an elaborate list of questions including all aspects of life, with only a subset on PA. Thus, the patients in our study might have overestimated their PA.

The limitations of this study are potential overestimation of outcome measures and recall bias, due to using the SQUASH questionnaire, more objective measures like accelerometers should be used in future studies. Furthermore, the preoperative levels of PA should be taken into account as an important confounder for outcome as well. Thus, more valid comparisons with the general population are possible. Also, patients in our study who refused to fill in the questionnaires were not asked about their reasons as to why they declined to participate, and therefore, we have no information about any possible self-selection bias. In addition, the comorbidities of participants were all self-reported and we were unable to confirm the presence of comorbidities both in the general Dutch population and the arthroplasty groups.

The findings of this study give insights into the movement patterns of arthroplasty patients compared to the general Dutch population. Findings show that although a part of the arthroplasty patients adhere to the Dutch public health guideline, there is still a considerable group who should increase their PA levels.

### Summary conclusion

Overall OA patients with a hip prosthesis have a higher level of activity compared to the general Dutch population when adjusted for age, sex, education and BMI.
